# A General Fuzzy Cerebellar Model Neural Network Multidimensional Classifier Using Intuitionistic Fuzzy Sets for Medical Identification

**DOI:** 10.1155/2016/8073279

**Published:** 2016-05-19

**Authors:** Jing Zhao, Lo-Yi Lin, Chih-Min Lin

**Affiliations:** ^1^School of Electrical Engineering & Automation, Xiamen University of Technology, Xiamen 361024, China; ^2^Department of Electrical Engineering, Yuan Ze University, Taoyuan 320, Taiwan; ^3^School of Medicine, Taipei Medical University, Taipei 100, Taiwan; ^4^Department of Electrical Engineering and Innovation Center for Big Data and Digital Convergence, Yuan Ze University, Taoyuan 320, Taiwan; ^5^School of Information Science and Engineering, Xiamen University, Xiamen 361005, China

## Abstract

The diversity of medical factors makes the analysis and judgment of uncertainty one of the challenges of medical diagnosis. A well-designed classification and judgment system for medical uncertainty can increase the rate of correct medical diagnosis. In this paper, a new multidimensional classifier is proposed by using an intelligent algorithm, which is the general fuzzy cerebellar model neural network (GFCMNN). To obtain more information about uncertainty, an intuitionistic fuzzy linguistic term is employed to describe medical features. The solution of classification is obtained by a similarity measurement. The advantages of the novel classifier proposed here are drawn out by comparing the same medical example under the methods of intuitionistic fuzzy sets (IFSs) and intuitionistic fuzzy cross-entropy (IFCE) with different score functions. Cross verification experiments are also taken to further test the classification ability of the GFCMNN multidimensional classifier. All of these experimental results show the effectiveness of the proposed GFCMNN multidimensional classifier and point out that it can assist in supporting for correct medical diagnoses associated with multiple categories.

## 1. Introduction

In most of the medical diagnosis problems, there exist some base patterns, and the medical decisions can be made on the basis of the similarity between the unknown samples and the base patterns [1–3]. Uncertainty is an inherent characteristic of medical problems [4, 5], so fuzzy approach could be appropriate to deal with these problems [6]. In order to describe uncertainty more accurately, a suitable fuzzy set is necessary.

Fuzzy sets (FSs), proposed by Zadeh [7], are frameworks to employ when encountering some vagueness. Based on the concepts of fuzzy set theory, numerous fuzzy approaches to medical diagnosis have been applied [8–12]. Another fuzzy theory, intuitionistic fuzzy sets (IFSs), has been widely used in several investigations of medical diagnosis [8, 13–16]. IFSs were proposed by the Bulgarian scholar Atanassov in 1986 [17]. On the basis of conventional fuzzy sets, a new attribute parameter, the nonmembership function, is added, which can describe the characterization of fuzzy nature more precisely. Due to the abundance of fuzzy linguistic terms in comparison to conventional fuzzy logic, interest in adopting IFSs with artificial neural networks has emerged [18–25]. The IFSs approach can combine the capability of intuitionistic fuzzy reasoning in handling uncertain information and the benefits of artificial learning in modeling the systems.

This paper proposed a novel multidimensional classifier based on an intelligent algorithm in IFSs. This method is referred to as a fuzzy cerebellar model neural network (FCMNN). A cerebellar model neural network (CMNN) can be thought of as a learning mechanism imitating the cerebellum of a human being and possesses a non-fully connected perceptron-like associative memory network with overlapping receptive fields [26]. It has already been shown to be able to approximate a nonlinear function over a domain of interest to any desired accuracy. Combined with fuzzy theory, FCMNN not only offers a unique and flexible framework for knowledge representation but also processes the quick learning ability of CMAC. The advantages of using FCMNN in many applications have been well documented [27–29], such as good generalization and rapid learning speed and convergence. Moreover, FCMNN can be viewed as the generation of a fuzzy neural network. If each layer of the FCMNN is reduced to contain only one different neuron, then it can be reduced to a fuzzy NN [30–33], such that it also can be called the general fuzzy cerebellar model neural network (GFCMNN). Therefore, a GFCMNN multidimensional classifier is designed for medical classification problems in IFSs with a similarity measure. Finally, some simulations and comparisons are performed to illustrate the effectiveness of the proposed design method.

This paper is organized as follows. The GFCMNN multidimensional classifier is introduced in Section 2.  Section 3 describes the medical data features in IFSs. In Section 4, experimental results are provided to illustrate the effectiveness of the proposed classifier. Finally, conclusions are drawn in Section 5.

## 2. General Fuzzy Cerebellar Model Neural Network Multidimensional Classifier

In most cases, a cerebellar model neural network is applied without fuzzy rules. To enable better use of experience knowledge, an extended general fuzzy cerebellar model neural network is designed for the multidimensional classifier.

### 2.1. Structure of the GFCMNN Multidimensional Classifier

A GFCMNN with the following fuzzy inference rules is proposed:

(1) Rλ:  If I1  is  f1jk,I2  is  f2jk,…,Ini  is  fnijk,then Oo=wjkofor  j=1,2,…,nj,  k=1,2,…,nk,  o=1,2,…,no,  λ=1,2,…,nl,

where *R* ^*λ*^ is the *λ*th rule, *n*_*i*_ is the input dimension, *n*_*j*_ is the number of the layers for each input dimension, *n*_*k*_ is the number of blocks for each layer, *n*_*o*_ is the output dimension, *n*_*l*_ = *n*_*j*_ *n*_*k*_ is the number of the fuzzy rules, *f*_*ijk*_ is the fuzzy set for the *i*th input, *j*th layer, and *k*th block, and *w*_*jko*_ is the weight for the *o*th output in the consequent part.

**Figure 1:**
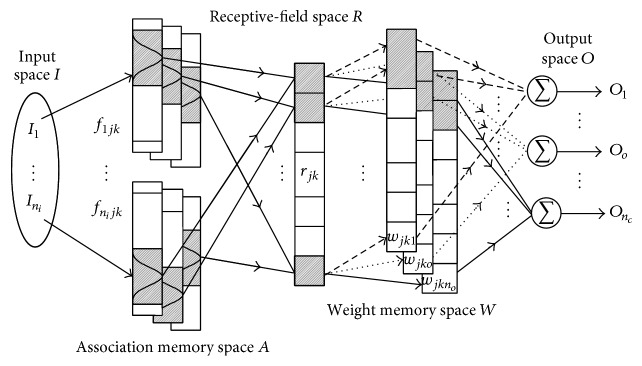
Architecture of a GFCMNN.

The architecture of this GFCMNN is shown in Figure 1. It is different from the fuzzy neural network (FNN) because the processing structure includes layers and blocks in the association memory space.

In this GFCMNN, if each layer is reduced to contain only one different neuron, then this GFCMNN can be reduced to an FNN. Thus, this GFCMNN can be viewed as a generalization of an FNN, and it offers better generalization, faster leaning, and quicker recall than the FNN.

The GFCMNN is composed of two consequent mappings and an output computation with the spaces of the input space *I*, association memory space *A*, receptive-field space *R*, weight memory space *W*, and output space *O*. These functional mappings are Mapping *I* : *I* → *A*, Mapping *A* : *A* → *R*, and Mapping *R* : *R* → *W*, where *I* and *O* are *n*_*i*_-dimension and *n*_*o*_-dimension, respectively.

The signal propagation and the basic function in each space are described as follows.

#### 2.1.1. Input: Input Space *I*

For a given **I** = [*I*_1_,…, *I*_*i*_,…, *I*_*n*_*i*__]^*T*^ ∈ *ℜ* ^*n*_*i*_^, each input state variable *I*_*i*_ is assumed to be quantized into *n*_*e*_ discrete regions (called “elements” or “neurons”). The number of elements *n*_*e*_ is termed as a resolution.

#### 2.1.2. Membership Function: Association Memory Space *A*

In this space, several elements can be accumulated as a block. The number of blocks *n*_*B*_, in the GFCMNN, is usually greater than two. By shifting each element, different blocks will be obtained. In this space, each block performs a receptive-field membership function. Here, the Gaussian function is adopted as the receptive-field membership function represented as

(2) fijk=exp⁡−Ii−mijk2vijk2,for  i=1,2,…,ni,  j=1,2,…,nj,  k=1,2,…,nk,

where *m*_*ijk*_ is the mean and *v*_*ijk*_ is the variance of the *j*th layer and *k*th block membership function corresponding to the *i*th input variable.

#### 2.1.3. Fuzzy Inference: Receptive-Field Space *R*

The product fuzzy inference is used as the “and” computation in the antecedent part. Thus, hypercubes, called receptive fields, are formed by multiple-input regions. The number of hypercubes is equal to *n*_*l*_ = *n*_*j*_ *n*_*k*_. The content of a hypercube can be expressed as *r*_*jk*_, which is the general basis function associated with the *j*th layer and *k*th block, that is

(3) rjk=∏i=1nifijk=−∏i=1niexp⁡−Ii−mijk2vijk2,for  j=1,2,…,nj,  k=1,2,…,nk.

The multidimensional receptive-field functions can be expressed in a vector form as

(4) rr11,…,r1nk,r21,…,r2nk,…,rnj1,…,rnjnkT∈Rnjnk.

#### 2.1.4. Fuzzy Output: Weight Memory Space *W*

Each location of the receptive field in relation to a particular adjustable value in the weight memory space can be expressed as

(5) wo=w11o,…,w1nko,w21o,…,w2nko,…,wnj1o,…,wnjnkoT∈Rnjnk,for  o=1,2,…,no,

where *w*_*jko*_ denotes the connecting weight value of the *o*th category output associated with the *j*th layer and *k*th block.

#### 2.1.5. Category Output: Output Space *O*

The GFCMNN output is the algebraic sum of the hypercube contents with activated weights. The *o*th output of the GFCMNN multidimensional classifier is represented as

(6) $$\begin{array}{c} O_{o}=w_{o}^{T}r=\overset{n_{j}}{\underset{j=1}{\sum }}\overset{n_{k}}{\underset{k=1}{\sum }}w_{jko}^{}r_{jk}^{},\;\text{for }o=1,2,\ldots ,n_{o}. \end{array}$$

### 2.2. Normalized Gradient Descent Algorithm

Because of the characteristic of fast convergence, the normalized iterative gradient decent algorithm is applied to adjust the parameters, and back propagation (BP) has been designed to deduce the learning rule of this GFCMNN multidimensional classifier.

#### 2.2.1. Cost Function

To describe the online learning algorithm of GFCMNN, the cost function *E* is defined as

(7) $$\begin{array}{c} E\left(\;k\;\right)=\frac{1}{2}\overset{n_{o}}{\underset{o=1}{\sum }}{\left(\;d_{o}\left(\;k\;\right)-O_{o}\left(\;k\;\right)\;\right)}^{2}=\frac{1}{2}\overset{n_{o}}{\underset{o=1}{\sum }}e_{o}^{2}\left(\;k\;\right), \end{array}$$

where *e*_*o*_(*k*) = *d*_*o*_(*k*) − *O*_*o*_(*k*) denotes the error of *o*th category output, *d*_*o*_(*k*) is the *o*th target output, and *O*_*o*_(*k*) is the *o*th category output of GFCMNN.

#### 2.2.2. Update Learning Laws

With the cost function *E*(*k*), the parameter updating learning law of GFCMNN based on the normalized gradient descent algorithm can be derived according to

(8) $$\begin{array}{c} z\left(\;k+1\;\right)=z\left(\;k\;\right)+\Delta z\left(\;k\;\right)=z\left(\;k\;\right)-\eta_{z}\frac{\partial E}{\partial O_{o}}P_{z}\left(\;k\;\right), \end{array}$$

where *z* is replaced by *w*, *m*, and *v*, denoting the updating law for output weight, mean, and variance, respectively. Moreover, the gradient operation factor **P**_*z*_(*k*) = ∂*O*_*o*_/∂*z* in (8) is defined as

(9) Pwk=∂Oo∂w=∂Oo∂w11o,…,∂Oo∂w1nko,∂Oo∂w21o,…,∂Oo∂w2nko,…,∂Oo∂wnj1o,…,∂Oo∂wnjnkoT,

(10) Pmk=∂Oo∂m=∂Oo∂mi11,…,∂Oo∂mi1nk,∂Oo∂mi21,…,∂Oo∂mi2nk,…,∂Oo∂minj1,…,∂Oo∂minjnkT,

(11) Pvk=∂Oo∂v=∂Oo∂vi11,…,∂Oo∂vi1nk,∂Oo∂vi21,…,∂Oo∂vi2nk,…,∂Oo∂vinj1,…,∂Oo∂vinjnkT.

Then, the parameter adjustment rules of weight, mean, and variance can be described as

(12) Δwjko=−ηw∂E∂wjko=−ηw∂∂yo·12do−Oo2∂∂wjkowjkorjk=ηweorjk,

(13) Δmijk=−ηm∑o=1no∂Ek∂mijk=−2ηm∑o=1no−do−Oowjko·exp−Ii−mijk2vijk2Ii−mijkvijk−2=2ηm∑o=1noeowjkorjkIi−mijkvijk−2,

(14) Δvijk=−ηv∑o=1no∂Ek∂vijk=−2ηv∑o=1no−do−Oowjko·exp−Ii−mijk2vijk2Ii−mijk2vijk−3=2ηv∑o=1noeowjkorjkIi−mijk2vijk−3,

where *η*_*w*_, *η*_*m*_, and *η*_*v*_ are the learning rates of output weight, mean, and variance, respectively.

### 2.3. Convergence Analyses

The learning laws in (12), (13), and (14) call for a proper choice of the learning rates *η*_*w*_, *η*_*m*_, and *η*_*v*_. For a small value of learning rates, the convergence is easy to guarantee; however, the learning speed is slow. On the other hand, if learning rates are too large, the learning mechanism may become more unstable. In order to train the GFCMNN effectively, the variable learning rates, which guarantee the convergence of the output error, are derived as follows. Moreover, the optimal learning rates which guarantee the fastest convergence of the output error are also derived.

**Theorem 1 .**

Let *η*_*z*_ be the learning rate of the GFCMNN, and let **P**_*z*_(*k*) be given in (9), (10), and (11) for *z* = *w*, *m*, or *v*, respectively. Then, the convergence of the tracking error is guaranteed if *η*_*z*_ is chosen as

(15) $$\begin{array}{c} 0<\eta_{z}<\frac{2}{{\left\|P_{z}\left(k\right)\right\|}^{2}}. \end{array}$$

Moreover, the variable optimal learning rate equals

(16) $$\begin{array}{c} \eta_{z}^{*}=\frac{1}{{\left\|P_{z}\left(k\right)\right\|}^{2}}. \end{array}$$

**Proof**

Define a Lyapunov function as

(17) $$\begin{array}{c} V\left(\;k\;\right)=\frac{1}{2}e_{o}^{2}\left(\;k\;\right). \end{array}$$

Then, the change of the Lyapunov function is obtained as

(18) $$\begin{array}{c} \Delta V\left(\;k\;\right)=V\left(\;k+1\;\right)-V\left(\;k\;\right)=\frac{1}{2}\left[\;e_{o}^{2}\left(\;k+1\;\right)-e_{o}^{2}\left(\;k\;\right)\;\right] \end{array}$$

and the error difference can be represented by

(19) $$\begin{array}{c} e_{o}\left(\;k+1\;\right)=e_{o}\left(\;k\;\right)+\Delta e_{o}\left(\;k\;\right)≅e_{o}\left(\;k\;\right)+{\left[\;\frac{\partial e_{o}\left(k\right)}{\partial z}\;\right]}^{T}\Delta z. \end{array}$$

Using the chain rule, the following is obtained:

(20) $$\begin{array}{c} \frac{\partial e_{o}\left(k\right)}{\partial z}=\frac{\partial e_{o}\left(k\right)}{\partial O_{o}\left(k\right)}\frac{\partial O_{o}\left(k\right)}{\partial z}=-P_{z}\left(\;k\;\right). \end{array}$$

Thus,

(21) ek+1ek−PzkTηzekPzk=ek1−ηzPzk2.

Substituting (21) into (18), Δ*V*(*k*) can be represented as

(22) $$\begin{array}{c} \Delta V\left(\;k\;\right)=\frac{1}{2}\eta_{z}e^{2}\left(\;k\;\right){\left\|\;P_{z}\left(\;k\;\right)\;\right\|}^{2}\left(\;\eta_{z}{\left\|\;P_{z}\left(\;k\;\right)\;\right\|}^{2}-2\;\right). \end{array}$$

If *η*_*z*_ is chosen as (15), Δ*V*(*k*) in (22) is less than 0. Therefore, the Lyapunov stability of *V* > 0 and Δ*V* < 0 is guaranteed. Thus, the convergence of tracking error *e*_*o*_(*k*) is guaranteed. Moreover, for the sake of achieving the fastest convergence, the optimal learning rates correspond to *η*_*z*_^*∗*^ = 1/‖**P**_*z*_(*k*)‖^2^, which comes from the derivative of (22) with respect to *η*_*z*_ and equals zero. This shows an interesting result, that is, variable optimal learning rates which can be adjusted online at each instant to achieve the fastest convergence of the tracking error with guaranteed stability.

In conclusion, the GFCMNN multidimensional classifier is defined by (6). The parameter learning rule is deduced by the normalized gradient descent algorithm and the weight, mean, and variance can be adjusted according to (12), (13), and (14), respectively. The optimal learning rates are designed to guarantee the convergence of this GFCMNN multidimensional classifier.

## 3. Medical Data Features in Intuitionistic Fuzzy Sets

### 3.1. Intuitionistic Fuzzy Sets for Medical Data

To make a proper medical classification, a medical knowledge base is necessary. In this paper, an IFS for a medical knowledge base is considered.

Fuzzy sets theory, proposed by Zadeh [7] in 1965, has been successfully applied in various fields. In this theory, the membership of an element to a fuzzy set is a single value between zero and one, and the linguistic variables are given in terms of a membership function only. However, in some situations, such as medical diagnosis, sales analysis, and financial services, which are described by a conventional fuzzy set, this theory seems too rough. Due to the possibility of a nonnull hesitation part for any unknown object at each moment when estimating, the degree of nonmembership of an element to a fuzzy set is just equal to 1 minus the degree of membership; that is, there may be some degree of hesitation. Thus, as a generalization of fuzzy sets, the concept of IFSs was introduced by Atanassov in 1986 [15].

In an intuitionistic fuzzy set, *A* in the universe of discourse *X* can be defined as a set of ordered pairs:

(23) $$\begin{array}{c} A=\left\{\;\langle \;x,\mu_{A}\left(\;x\;\right),v_{A}\left(\;x\;\right)\;\rangle \;|\;x\in X\;\right\}, \end{array}$$

where *μ*_*A*_: *X* → [0,1] and *v*_*A*_: *X* → [0,1] indicate the degree of *x* which belongs to *A* and does not belong to *A*, respectively. *μ*_*A*_(·) is called the membership function, and *v*_*A*_(·) is called the nonmembership function.

For each IFS *A* in *X*, the “hesitation margin” (or “intuitionistic fuzzy index”) of *x* ∈ *X* is given by

(24) $$\begin{array}{c} \pi_{A}\left(\;x\;\right)=1-\mu_{A}\left(\;x\;\right)-v_{A}\left(\;x\;\right), \end{array}$$

where 0 ≤ *μ*_*A*_(*x*) ≤ 1, 0 ≤ *v*_*A*_(*x*) ≤ 1, and 0 ≤ *π*_*A*_(*x*) ≤ 1, ∀*x* ∈ *X*, which expresses the hesitation degree of whether *x* belongs to *A* or not.

**Figure 2:**
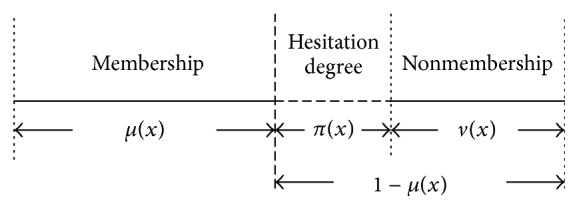
Descriptions of the IFSs.

An illustration of these degrees is exhibited in Figure 2. Consequently, IFSs are an extension of the conventional FSs. To describe an IFS completely, at any rate, two functions are needed, one being the membership function and the other being the nonmembership function. In the aspects of semantic expression and reasoning ability, this is clearly better than conventional FSs.

In the case of medical diagnosis, we consider the same IFSs as in the disease classification in [34, 35]. There are five diseases in Table 1 to build the set of diseases *D* = [viral fever, malaria, typhoid, stomach problem, chest pain]. Each disease has five symptoms to form the set of features *F* = [temperature, headache, stomach pain, cough, chest pain]. Each element in the table is given in the form of a group of numbers corresponding to the membership, nonmembership, and hesitation values, respectively. For example, the temperature for viral fever is described by (*μ*, *v*, *π*) = (0.4,0.0,0.6) in Table 1.

### 3.2. Score Function of IFSs

In the traditional FSs, the fuzzy relationship is obtained by the max-min-max composition. In order to fully use the provided information of IFSs, the intuitionistic fuzzy relation can be described by use of the score function, which is usually used to judge the matching degree between the intuitionistic fuzzy relation and the decision requirements.

The score function, such as *R* = *μ* − *v*, which is proposed by Chen and Tan [36], has two elements of IFSs, but another element of hesitation degree *π* cannot be taken into account.

A modification of the score function has been proposed (score function 1) [37]:

(25) $$\begin{array}{c} R=\mu -v\pi . \end{array}$$

Despite considering the factor of *π*, the proportion of the membership function *μ* has actually been weakened because of adopting the subtracted form. To overcome this shortcoming, another added form is defined as (score function 2) [38]

(26) $$\begin{array}{c} R=\mu +v\pi . \end{array}$$

However, in fact, a reasonable description of IFSs would be as follows: the higher the proportion of the membership function, the lesser the proportion of the nonmembership function, that is, the maximum difference between *μ* and *v* with the minimum hesitation degree *π*. Therefore, to describe the IFSs relation much more accurately and carefully, the applied score function is defined as (score function 3)

(27) $$\begin{array}{c} R=\mu -v+\frac{1}{\alpha \pi +\beta }, \end{array}$$

where *α* and *β* are the constants. The greater the value of *R* is, the more accordant to the reality the event will be, which is described by (27) with the diverse values of *α* and *β*. By using this kind of score function, the different decision-making degrees of the three members in the IFSs are embodied while handling uncertain information, such that not only have the shortcomings of the above score functions been made up, but also the ability of the fuzzy linguistic expression has become more precise, and the description in modeling a system is more complete.

## 4. Experiment and Discussion

### 4.1. Similarity Measure for Multidimensional Classifier

The solutions of the classification are usually the distance measure and the similarity measure. Distance is defined as a quantitative degree of how far apart two objects are, while similarity is defined as the degree of similarity between two sets. In this paper, the similarity measure is used as a performance index to discuss the classification results.

The similarity measure of the algorithm is defined as follows:

(28) $$\begin{array}{c} P_{\mathrm{erf}}=\frac{\left|A\cap B\right|}{\left|A\cup B\right|}, \end{array}$$

where *A* is the target data set, *B* is the testing data set, ∩ and ∪ denote the intersection and union of *A* and *B*, respectively, and |·| indicates the cardinality of a set. This relative cardinality evaluates the proportion of elements of *A* ∪ *B* having the property *A*∩*B*, when *A* ∪ *B* is finite.

### 4.2. Medical Diagnosis Example

The medical diagnosis classification trained data are shown in Table 1. The tested data are shown in Table 2, which consists of a set of patients *S* = [Al, Bob, Joe, Ted]. The symptoms for each patient are also given in Table 2.

Five features of the diseases are taken as the input of GFCMNN; that is, the input consists of 5 dimensions. And the given data shown in Table 1 are used to train the proposed GFCMNN multidimensional classifier offline. It should be classified into 5 categories according to the five diseases. In other words, the output dimension of GFCMNN is 5. Then, the considered data shown in Table 2 are used to test the correctness of this classifier.

In this case, the GFCMNN is characterized as *n*_*e*_ = 5 for each input dimension to cover the input range with enough resolution; then, four layers are used and every layer contains five neurons. In the initial parameters setting, the learning rates of the GFCMNN classifier are 0.1. All of the parameters are determined by trial-and-error, in order to guarantee the desired classification performance. The initial weights, means, and variances, *w*_*jko*_, *m*_*ijk*_, and *v*_*ijk*_, are generated randomly.

The sample types of the medical IFSs are set as membership function *μ*, (25), (26), and (27), where *α* = 100 and *β* = 10. The tested performances of GFCMNN are shown in Tables 3, 4, 5, and 6.

According to the tested performances from Tables 3 [Table tab4] [Table tab5]–6, if only the membership function *μ* is used, the classification results are as follows: Al suffers from malaria, Bob from typhoid, Joe from typhoid, and Ted from malaria. If the score function (25) is used as the type of input, the classification results are as follows: Al suffers from malaria, Bob from typhoid, Joe from typhoid, and Ted from viral fever. When the score function (26) is used as the type of input, the classification results are as follows: Al suffers from malaria, Bob from stomach problem, Joe from typhoid, and Ted from viral fever. Under the score function (27), with *α* and *β* values identical to those above used as the pattern of input, the same classified results are obtained. Meanwhile the doctor's diagnosis results are as follows: Al suffers from malaria, Bob from stomach problem, Joe from typhoid, and Ted from malaria.

Compared to the same example, a type of score function, like (25), is used in [27], and the max-min-max rule is applied to classify the five categories. Another kind of score function like *μ* + *v* was adopted, in [39], which used the method of intuitionistic fuzzy cross-entropy (IFCE) to adjust the same medical diagnosis. After mapping these five diseases to class 1–class 5, the results of comparison are tabulated in Table 7. The doctor's diagnosis is also given for comparison.

Table 7 shows that these disease samples can be classified by using the GFCMNN multidimensional classifier, and the accuracy is much better than those of the IFSs classifier and the IFCE classifier, even with the use of an input pattern, like *μ*. With the same input pattern, the classification precision of the GFCMNN classifier is also superior to that of the IFSs classifier. After adopting the GFCMNN input patterns, such as (26) and (27), the classification results are consistent with the doctor's diagnosis, although the input pattern, for instance (26), is not the best input pattern for intuitionistic fuzzy data.

### 4.3. Cross Validation

To further test the classification ability of the GFCMNN multidimensional classifier, the cross verification method is adopted, after combining the 5 trained samples with the 4 tested samples, which were correctly classified previously, and renumbering them as shown in Table 8.

These renumbered samples are divided into 2 groups: the trained set with 5 samples and the tested set with 4 samples. In the cross validation phase, one sample of the same type is exchanged for each round by using the GFCMNN input pattern, such as membership function *μ*, (26) and (27). The initial parameters of GFCMNN and the score function 3 are set the same as previously mentioned. The test results are shown in Tables 9 [Table tab10]–11.

In this case, from Tables 9 [Table tab10]–11, the true results are *T* = 16, *T* = 18, and *T* = 19, while the wrong results are *N* = 4, *N* = 2, and *N* = 1. Thus, the accuracy is equal to 80%, 90%, and 95%, as calculated by

(29) $$\begin{array}{c} \text{ACC}=\frac{T}{T+N}\times 100\%. \end{array}$$

The experimental results show that multiple type medical data can be identified by the proposed GFCMNN classifier. Moreover, combined with fuzzy intuitionistic data, much better classification precision has been achieved.

## 5. Conclusion

The proposed GFCMNN multidimensional classifier is an extended structure composed of a fuzzy system and CMNN, with the respective advantages of each. The classification efficiency can be improved because of the better generalization ability, learning ability, and approximate ability of the proposed approach. When combined with the IFSs, the original features can be better presented and the classification accuracy is also enhanced. Meanwhile, the experimental results have demonstrated the effectiveness of the proposed classifier. Therefore, the classification results of the GFCMNM multidimensional classifier can assist doctors by supporting the medical diagnosis.

## Figures and Tables

**Table 1:** Feature values for the studied diseases categories.

Disease	Feature				
	Temperature	Headache	Stomach pain	Cough	Chest pain
Viral fever	(0.4, 0.0, 0.6)	(0.3, 0.5, 0.2)	(0.1, 0.7, 0.2)	(0.4, 0.3, 0.3)	(0.1, 0.7, 0.2)
Malaria	(0.7, 0.0, 0.3)	(0.2, 0.6, 0.2)	(0.0, 0.9, 0.1)	(0.7, 0.0, 0.3)	(0.1, 0.8, 0.1)
Typhoid	(0.3, 0.3, 0.4)	(0.6, 0.1, 0.3)	(0.2, 0.7, 0.1)	(0.2, 0.6, 0.2)	(0.1, 0.9, 0.0)
Stomach problem	(0.1, 0.7, 0.2)	(0.2, 0.4, 0.4)	(0.8, 0.0, 0.2)	(0.2, 0.7, 0.1)	(0.5, 0.7, 0.1)
Chest problem	(0.1, 0.8, 0.1)	(0.0, 0.8, 0.0)	(0.2, 0.8, 0.0)	(0.2, 0.8, 0.0)	(0.8, 0.1, 0.1)

**Table 2:** Data set for the studied diseases categories.

Sample	Feature				
	Temperature	Headache	Stomach pain	Cough	Chest pain
Al	(0.8, 0.1, 0.1)	(0.6, 0.1, 0.3)	(0.2, 0.8, 0.0)	(0.6, 0.1, 0.3)	(0.1, 0.6, 0.3)
Bob	(0.0, 0.8, 0.2)	(0.4, 0.4, 0.2)	(0.6, 0.1, 0.3)	(0.1, 0.7, 0.2)	(0.1, 0.8, 0.1)
Joe	(0.8, 0.1, 0.1)	(0.8, 0.1, 0.1)	(0.0, 0.6, 0.4)	(0.2, 0.7, 0.1)	(0.0, 0.5, 0.5)
Ted	(0.6, 0.1, 0.3)	(0.5, 0.4, 0.1)	(0.3, 0.4, 0.3)	(0.7, 0.2, 0.1)	(0.3, 0.4, 0.3)

**Table 3:** Test performances of GFCMNN with membership function.

Sample	Disease				
	Viral fever	Malaria	Typhoid	Stomach problem	Chest problem
Al	0.4950	0.6934	0.4761	0.1916	0.1480
Bob	0.3555	0.1897	0.6474	0.3890	0.2129
Joe	0.5384	0.4498	0.5141	0.1368	0.0769
Ted	0.4594	0.6007	0.3908	0.3951	0.2139

**Table 4 tab4:** Test performances of GFCMNN with score function 1.

Sample	Disease				
	Viral fever	Malaria	Typhoid	Stomach problem	Chest problem
Al	0.2667	0.5675	0.1070	0.1299	0.0143
Bob	0.2274	0.0971	0.7885	0.1009	0.0446
Joe	0.1277	0.0584	0.2342	0.0506	0.0388
Ted	0.2346	0.4032	0.1702	0.1562	0.0212

**Table 5 tab5:** Test performances of GFCMNN with score function 2.

Sample	Disease				
	Viral fever	Malaria	Typhoid	Stomach problem	Chest problem
Al	0.7045	0.8052	0.6539	0.4150	0.2902
Bob	0.5600	0.3649	0.6441	0.6847	0.3993
Joe	0.6839	0.5995	0.8315	0.4405	0.3013
Ted	0.6430	0.6462	0.5631	0.6305	0.2810

**Table 6:** Test performances of GFCMNN with score function 3.

Sample	Disease				
	Viral fever	Malaria	Typhoid	Stomach problem	Chest problem
Al	0.5581	0.6776	0.3011	0.0452	0.1422
Bob	0.4679	0.1940	0.6461	0.6862	0.3090
Joe	0.7798	0.5253	0.8112	0.4288	0.2623
Ted	0.4274	0.6032	0.1596	0.0939	0.2586

**Table 7:** Comparing the test performances of GFCMNN with score function 3.

Sample	GFCMNN				IFS *μ* − *vπ*	IFCE *μ* + *v*	Doctor
	*μ*	*μ* − *vπ*	*μ* + *vπ*	*μ* − *v* + 1/(*απ* + *β*)			
Al	2	2	2	2	2	1	2
Bob	3	3	4	4	3	4	4
Joe	1	3	3	3	2	1	3
Ted	2	2	2	2	2	1	2

**Table 8:** Renumbering of the medical intuitionistic fuzzy sample.

Sample number	Original info	Category
1	Viral fever	1
2	Malaria	2
3	Typhoid	3
4	Stomach problem	4
5	Chest problem	5
6	Ted	1
7	Al	2
8	Joe	3
9	Bob	4

**Table 9:** Cross validation results of GFCMNN with membership function 1.

Sample	Test 1	Test 2	Test 3	Test 4	Test 5	Expected
s1	2	2	2	2	2	2
s2	2	2	2	2	2	2
s3	1	3	3	3	3	3
s4	3	4	3	3	4	4

**Table 10 tab10:** Cross validation results of GFCMNN with score function 2.

Sample	Test 1	Test 2	Test 3	Test 4	Test 5	Expected
s1	2	2	2	2	2	2
s2	2	2	2	2	2	2
s3	3	3	3	3	3	3
s4	4	4	3	4	3	4

**Table 11:** Cross validation results of GFCMNN with score function 3.

Sample	Test 1	Test 2	Test 3	Test 4	Test 5	Expected
s1	2	2	2	2	2	2
s2	2	2	2	2	2	2
s3	3	3	3	3	3	3
s4	4	4	4	3	4	4

## References

[1] Wolff J. G. (2006). Medical diagnosis as pattern recognition in a framework of information compression by multiple alignment, unification and search. *Decision Support Systems*.

[2] Sanz J. A., Galar M., Jurio A., Brugos A., Pagola M., Bustince H. (2014). Medical diagnosis of cardiovascular diseases using an interval-valued fuzzy rule-based classification system. *Applied Soft Computing Journal*.

[3] Soni J., Ansari U., Sharma D., Soni S. (2011). Predictive data mining for medical diagnosis: an overview of heart disease prediction. *International Journal of Computer Applications*.

[4] Straszecka E. (2006). Combining uncertainty and imprecision in models of medical diagnosis. *Information Sciences*.

[5] Han J. W., Kamber M. (2006). *Data Mining: Concepts and Techniques*.

[6] Vlachos I. K., Sergiadis G. D. (2007). Intuitionistic fuzzy information: applications to pattern recognition. *Pattern Recognition Letters*.

[7] Zadeh L. A. (1965). Fuzzy sets. *Information and Computation*.

[8] Badaloni S., Falda M. (2010). Temporal-based medical diagnoses using a fuzzy temporal reasoning system. *Journal of Intelligent Manufacturing*.

[9] Lekkas S., Mikhailov L. (2010). Evolving fuzzy medical diagnosis of Pima Indians diabetes and of dermatological diseases. *Artificial Intelligence in Medicine*.

[10] Keleş A., Keleş A., Yavuz U. (2011). Expert system based on neuro-fuzzy rules for diagnosis breast cancer. *Expert Systems with Applications*.

[11] Amato F., López A., Peña-Méndez E. M., Vaňhara P., Hampl A., Havel J. (2013). Artificial neural networks in medical diagnosis. *Journal of Applied Biomedicine*.

[12] Papageorgiou E. I. (2011). A new methodology for decisions in medical informatics using fuzzy cognitive maps based on fuzzy rule-extraction techniques. *Applied Soft Computing*.

[13] Own C.-M. (2009). Switching between type-2 fuzzy sets and intuitionistic fuzzy sets: an application in medical diagnosis. *Applied Intelligence*.

[14] Ye J. (2011). Cosine similarity measures for intuitionistic fuzzy sets and their applications. *Mathematical and Computer Modelling*.

[15] Shannon A., Kim S., Kim Y., Sorsich J., Atanassov K., Georgiev P. (1997). A possibility for implementation of elements of the intuitionistic fuzzy logic in decision making in medicine. *Notes on Intuitionistic Fuzzy Sets*.

[16] Todorova L., Atanassov K., Hadjitodorov S., Vassilev P. (2007). On an intuitionistic fuzzy approach for decision making in medicine: part 2. *Bioautomation*.

[17] Atanassov K. T. (1986). Intuitionistic fuzzy sets. *Fuzzy Sets and Systems*.

[18] Lei Y.-J., Lu Y.-L., Li Z.-Y. (2007). Function approximation capabilities of intuitionistic fuzzy reasoning neural networks. *Control and Decision*.

[19] Li L., Yang J., Wu W. (2011). Intuitionistic fuzzy hopfield neural network and its stability. *Neural Network World*.

[20] Hadjyisky L., Atanassov K. (1993). Intuitionistic fuzzy model of a neural network. *Busefal*.

[21] Sotirov S. (2007). Intuitionistic fuzzy estimations for connections of the transmit routines of the bluetooth interface. *Advanced Studies in Contemporary Mathematics*.

[22] Sotirov S., Atanassov K. (2009). Intuitionistic fuzzy feed forward neural network. *Cybernetics and Information Technologies*.

[23] Sotirov S., Sotirova E., Orozova D. (2009). Neural network for defining intuitionistic fuzzy sets in e-learning. *Notes on Intuitionistic Fuzzy Sets*.

[24] Vardeva I., Sotirov S. Intuitionistic fuzzy estimation of damaged packets with multilayer perceptron.

[25] Sotirov S., Dimitrov A. (2010). Neural network for defining intuitionistic fuzzy estimation in petroleum recognition. *Issues in Intuitionistic Fuzzy Sets and Generalized Nets*.

[26] Albus J. S. (1975). A new approach to manipulator control: the cerebellar model articulation controller (CMAC). *Journal of Dynamic Systems, Measurement, and Control*.

[27] Lin C.-M., Peng Y.-F. (2004). Adaptive CMAC-based supervisory control for uncertain nonlinear systems. *IEEE Transactions on Systems, Man, and Cybernetics, Part B: Cybernetics*.

[28] Lin C.-M., Chen L.-Y., Yeung D. S. (2010). Adaptive filter design using recurrent cerebellar model articulation controller. *IEEE Transactions on Neural Networks*.

[29] Lin C.-M., Li H.-Y. (2012). TSK fuzzy CMAC-based robust adaptive backstepping control for uncertain nonlinear systems. *IEEE Transactions on Fuzzy Systems*.

[30] Hsu C.-F., Lin C.-M., Yeh R.-G. (2013). Supervisory adaptive dynamic RBF-based neural-fuzzy control system design for unknown nonlinear systems. *Applied Soft Computing*.

[31] Lin C.-M., Ting A.-B., Hsu C.-F., Chung C.-M. (2012). Adaptive control for MIMO uncertain nonlinear systems using recurrent wavelet neural network. *International Journal of Neural Systems*.

[32] Lee C.-H., Chang F.-Y., Lin C.-M. (2014). An efficient interval type-2 fuzzy CMAC for chaos time-series prediction and synchronization. *IEEE Transactions on Cybernetics*.

[33] Lin C.-M., Li H.-Y. (2015). Dynamic Petri fuzzy cerebellar model articulation controller design for a magnetic levitation system and a two-axis linear piezoelectric ceramic motor drive system. *IEEE Transactions on Control Systems Technology*.

[34] De S. K., Biswas R., Roy A. R. (2001). An application of intuitionistic fuzzy sets in medical diagnosis. *Fuzzy Sets and Systems*.

[35] Szmidt E., Kacprzyk J. (2001). Intuitionistic fuzzy sets in some medical applications. *The 7th Fuzzy Days Dortmund*.

[36] Chen S. M., Tan J. M. (1994). Handling multicriteria fuzzy decision-making problems based on vague set theory. *Fuzzy Sets and Systems*.

[37] Szmidt E., Kacprzyk J., Alexandrov V. N., Dongarra J. J., Juliano B. A., Renner R. S., Tan C. J. K. (2001). Intuitionistic fuzzy sets in intelligent data analysis for medical diagnosis. *Computational Science—ICCS 2001*.

[38] Liu H. W. (2004). Vague set methods of multicriteria fuzzy decision making. *Systems Engineering—Theory & Practice*.

[39] Hung K.-C. (2012). Medical pattern recognition: applying an improved intuitionistic fuzzy cross-entropy approach. *Advances in Fuzzy Systems*.

